# The S1P/S1P1 Signaling Axis Plays Regulatory Functions in the Crosstalk Between Brain-Metastasizing Melanoma Cells and Microglia

**DOI:** 10.3390/cancers17193175

**Published:** 2025-09-29

**Authors:** Orit Adir, Orit Sagi-Assif, Shlomit Ben-Menachem, Isaac P. Witz, Sivan Izraely

**Affiliations:** 1 The Shmunis School of Biomedicine and Cancer Research, George S. Wise Faculty of Life Science, Tel Aviv University, Tel Aviv 6997801, Israel; oritadir@mail.tau.ac.il (O.A.); oritsa@tauex.tau.ac.il (O.S.-A.); benmena@tauex.tau.ac.il (S.B.-M.); isaacw@tauex.tau.ac.il (I.P.W.); 2 Department of Developmental Biology and Cancer Research, Institute of Medical Research—Israel-Canada, The Hebrew University of Jerusalem, Jerusalem 9112102, Israel

**Keywords:** melanoma, microglia, brain metastasis, S1P1, tumor microenvironment

## Abstract

Background/Objectives: The interaction between brain-metastasizing melanoma cells and surrounding microglia shapes the immune tumor microenvironment and influences tumor progression. Gene expression analysis revealed that sphingosine-1-phosphate receptor 1 (*S1PR1*), encoding the S1P1 receptor, is upregulated in microglia upon interaction with melanoma cells. Here, we investigated the functions of S1P1 in microglia and its contribution to melanoma–microglia crosstalk. Methods: We examined the effects of S1P1 inhibition on microglia and four brain-metastasizing human melanoma cell lines in monocultures and co-cultures using the selective S1P1 antagonist NIBR0213 and *S1PR1* gene knockdown. Results: We found that melanoma-secreted IL-6 upregulated *S1PR1* expression in microglia. S1P1 inhibition increased expression of CD32, CD150, and CD163 in microglia; however, CD150 and CD163 upregulation was abolished in the presence of melanoma cells. S1P1 inhibition downregulated immunosuppressive and anti-inflammatory factors in microglia, including *CD274*, *SOCS3*, *TGFBR1*, *TGFBR2*, and JunB, promoting a pro-inflammatory phenotype. It also reduced viability of both melanoma and microglia cells, inducing apoptosis in melanoma-associated microglia, possibly via downregulation of CH25H, an upstream regulator of SREBPs. In co-cultures, melanoma cells were more sensitive than microglia to NIBR0213-induced growth arrest. In 3D spheroid cultures, NIBR0213 delayed melanoma–microglia aggregation. Combined treatment with the BRAF inhibitor Vemurafenib and NIBR0213 enhanced Vemurafenib efficacy in three of four melanoma lines. Conclusions: S1P1 contributes to the immunosuppressive phenotype of microglia. Inhibiting the S1P/S1P1 axis impairs viability and crosstalk between melanoma cells and tumor-activated microglia, offering a potential therapeutic strategy for melanoma brain metastases.

## Simple Summary

Brain metastases are a frequent and lethal complication of melanoma. The tumor microenvironment is well recognized as a critical driver of cancer progression. Within the brain, melanoma cells engage in dynamic interactions with microglia, the resident immune cells. However, the mechanisms governing this crosstalk remain poorly understood. In our previous work, we showed that brain-metastasizing melanoma cells reprogram microglia, inducing molecular and functional changes, including upregulation of sphingosine-1-phosphate receptor 1 (*S1PR1*). Here we aimed to investigate whether this upregulation affects microglial phenotype and thereby contributes to microglia-mediated support of melanoma brain metastasis progression. We found that inhibition of S1P1 shifted microglia toward an anti-tumor state, which can facilitate infiltration of cytotoxic immune cells, and also attenuated the growth of both microglia and melanoma cells. These findings highlight the S1P1 pathway as a key player in the tumor–brain environment and suggest that its targeting could be a novel therapeutic approach against brain-metastasizing melanoma.

## 1. Introduction

Melanoma is among the most aggressive cancers, with a high propensity to metastasize to the brain. During the course of their disease, up to 40–60% of patients with advanced melanoma develop brain metastases, which are associated with poor prognosis and limited therapeutic options [1].

Despite advances in targeted therapies and immune checkpoint inhibitors, survival outcomes for melanoma patients with brain metastases remain dismal, highlighting the urgent need to identify new mechanisms that drive tumor progression in the brain and to uncover therapeutic targets within this context [2].

The brain microenvironment plays a crucial role in shaping metastatic colonization and growth. Within this niche, melanoma cells establish a complex crosstalk with resident non-cancerous cells, particularly microglia, the brain’s innate immune cells. Similar to the concept that the crosstalk between cancer and non-cancerous cells in the tumor microenvironment can either promote or block cancer progression [3,4], microglia are highly plastic and can acquire tumor-supportive or tumor-suppressive phenotypes depending on the surrounding signals. Increasing evidence indicates that melanoma cells exploit microglial functions to promote immune evasion, tumor growth, and invasion [5]. However, the molecular mechanisms that orchestrate this melanoma–microglia interplay remain incompletely understood.

Sphingosine-1-phosphate (S1P) is a bioactive lipid mediator that regulates diverse cellular processes including proliferation, survival, migration, and angiogenesis [6]. These effects are largely mediated through its transmembrane receptors (S1PR1–5), which are differentially expressed across tissues and immune cell subsets. Among them, sphingosine-1-phosphate receptor 1 (S1P1, encoded by *S1PR1*) is of a particular interest due to its central role in modulating immune cell trafficking and inflammatory responses [6,7,8]. For example, S1P regulates lymphocyte egress from the spleen and lymph nodes into the circulation, thus playing a key role in the inflammatory processes [6].

While the S1P–S1P1 axis is well-characterized in lymphoid cells [9], its function in myeloid cells remains less defined. In an experimental autoimmune encephalomyelitis (EAE) model, S1P1 activated and enhanced myeloid cell antigen presentation [7]. Conversely, S1P has also been reported to induce an anti-inflammatory phenotype in macrophages [8].

The sphingolipid sphingosine-1-phosphate (S1P) and its receptor 1 (S1P1) are critically involved in tumor progression across various cancer types, exerting their influence on both malignant cells and the tumor microenvironment [10].

In tumor cells, the normal regulation of S1P levels is disturbed, leading to elevated concentrations that promote tumor growth [11]. The tumor-promoting effects of S1P are often mediated through its G protein-coupled receptors, including S1P1 [12].

In breast cancer, the S1P/S1P1 axis plays a central role. S1P stimulates angiogenesis and lymphangiogenesis. S1P1 signaling within macrophages, triggered by S1P, fosters a tumor-promoting, anti-inflammatory, and proangiogenic phenotype. Moreover, STAT3-induced S1P1 expression is crucial for the persistent activation of STAT3 in tumor and inflammatory cells, thereby driving tumor progression [12,13]. In murine melanoma, S1P transmits oncogenic signals via S1P1-5 receptors present on both malignant and neighboring cells. Inhibition of S1P1 induced melanoma cell apoptosis in vitro, limited pulmonary metastasis development, and modulated the immune system in vivo. Specifically, melanoma cells overexpressing S1P1 demonstrate increased cell migration, and S1P regulates lymphocyte traffic via S1P1 [11,14].

S1P is also present at high concentrations in the brain [15]. Emerging evidence highlights the role of the S1P/S1P1 axis in brain tumor development and neuroinflammation, with particular emphasis on microglial activation. S1P, secreted by glioblastoma cells, actively recruits TAMs to the tumor site by S1PR signaling, involving the modulation of Rac1/RhoA, resulting in increased infiltration into the tumor, and a pro-tumorigenic, anti-inflammatory phenotype. Blocking S1PRs on TAMs restored their pro-inflammatory phenotype [15].

While the established role of S1P in modulating the tumor microenvironment and immune cell behavior in primary brain tumors suggests potential relevance, studies on the S1P/S1P1 axis in brain metastases are limited.

Our previous studies [16,17,18] demonstrated that brain-metastasizing melanoma cells (BMMCs) induce profound molecular and functional reprogramming of microglia, including upregulation of *S1PR1* expression. Given that S1P1 can drive both immunosuppressive and tumor-supportive phenotypes, and that microglia are key players in the response of brain components to metastatic cells, we hypothesized that S1P signaling influences microglial interactions with tumor cells, affecting metastasis progression.

In the present study, we investigated the role of S1P/S1P1 in the interaction between BMMCs and microglia. We asked whether melanoma-derived factors regulate microglial S1P1 expression, and whether pharmacological or genetic inhibition of S1P1 could shift microglial responses toward anti-tumor functions. Furthermore, we examined the impact of S1P1 inhibition on melanoma viability, tumor–microglia aggregation, and therapeutic sensitivity. Our findings reveal that targeting the S1P/S1P1 axis reprograms microglia into an anti-tumor state, suppresses melanoma growth, and enhances sensitivity to BRAF inhibition, highlighting this pathway as a promising therapeutic target in melanoma brain metastasis.

## 2. Materials and Methods

### 2.1. Cell Culture

Human brain-metastasizing melanoma cell (BMMC) variants YDFR.CB3, DP.CB2, M12.CB3, and M16.CB3 were previously established in our lab from the parental human cell lines YDFR (kindly provided by Prof. Michael Micksche, Department of Applied and Experimental Oncology, Vienna University, Austria) and from DP-0574-Me, UCLA-SO-M12, and UCLA-SO-M16 (kindly provided by Dr. Dave S.B. Hoon, Department of Translational Molecular Medicine and Sequencing Center, Saint John’s Cancer Institute at Providence Saint John’s Health Center, Santa Monica, CA, USA) [17,18]. In short, the human brain metastatic variants, CB2 or CB3, were produced by two or three cycles, respectively, of in vivo selection, as previously described [17]. Melanoma cells were maintained in RPMI-1640 medium (Sartorius AG, Göttingen, Germany) supplemented with 10% fetal calf serum (FCS), 2 mmol/mL L-glutamine, 100 units/mL penicillin, 0.1 mg/mL streptomycin, and 12.5 units/mL nystatin. Immortalized human microglia-SV40 HMC3 cells (ATCC, Manassas, VA, USA) were maintained in EMEM medium (Sartorius AG) supplemented with 10% FCS, 2 mmol/mL L-glutamine, 100 units/mL penicillin, 0.1 mg/mL streptomycin, and 12.5 units/mL nystatin. Human embryonic kidney 293T cell line (ATCC) was maintained in DMEM medium supplemented with 10% FCS, 2 mmol/mL L-glutamine, 100 units/mL penicillin, 0.1 mg/mL streptomycin, and 12.5 units/mL nystatin. A mixture of RPMI-1640 and EMEM culture mediums (1:1) supplemented with 0.5% FCS was used for all experiments with starvation medium. Cells were routinely cultured in an incubator with humidified air with 5% CO2 at 37 °C. Cell cultures were routinely tested (every 3 months) for Mycoplasma contamination using Mycoplasma Detection Kit-MycoStrip™ (rep-mys-10, Invitrogene, Carlsbad, CA, USA). Cell authentication by STR was performed periodically to validate cell identity. Cells were cultivated for up to two months, then new batches were thawed and utilized. To produce mCherry-expressing BMMCs, cells were transduced with a pQCXIP–mCherry plasmid (Clontech Laboratories, Inc., Mountain View, CA, USA), as previously described [18,19].

### 2.2. Preparation of Melanoma-Conditioned Medium

Melanoma-conditioned medium was obtained from cultured melanoma cells that were starved in 0.5% FCS-containing medium for 48 h. The conditioned medium was then collected, centrifuged for 5 min at 220× *g*, and filtered (0.45 μm, Whatman GmbH, Dassel, Germany).

### 2.3. Cytokines

Microglia cells were starved for 1 h prior to stimulation with cytokines for an additional 3 h. The cytokines applied included LIF (25 ng/mL), OSM (50 ng/mL), IL-6 (20 ng/mL), IL-11 (50 ng/mL), PDGF-AA (20 ng/mL), and TGF-β1 (10 ng/mL) (PeproTeck, Rocky Hill, NJ, USA).

### 2.4. IL-6Rα Inhibition Assay

Microglia cells were pre-treated for 1 h with IL-6Rα-blocking antibody (1 μg/mL, MAB227, R&D Systems, Minneapolis, MA, USA) or IgG1 isotype control antibody (1 μg/mL, MAB002, R&D Systems), then treated with MCM and anti-IL-6Rα/IgG1 isotype control for an additional 3 h.

### 2.5. RNA Isolation and Reverse Transcription Quantitative Real-Time PCR (RT-qPCR)

Isolation of total RNA was performed with TRI Reagent (T9424, Sigma-Aldrich, St. Louis, MO, USA). cDNA was prepared from RNA samples using the qScript cDNA Synthesis Kit (Quantabio, Beverly, MA, USA) according to the manufacturer’s instructions. Amplification reactions were performed with SYBR Green I (Thermo Fisher Scientific, Bedford, MA, USA) in triplicate in a CFX ConnectTM Real-Time System (Bio-Rad Laboratories, Hercules, CA, USA). PCR amplification was performed over 35–40 cycles (95 °C for 15 s, 59 °C for 20 s, and 72 °C for 15 s). Primer sequences are detailed below: CCL2: S-5′-TGCAATCAATGCCCCAGTCAC-3′, AS-5′-ACTTCTGCTTGGGGTCAGCAC-3′; CD274: S-5′-ATGACCTACTGGCATTTGCTA-3′, AS-5′-TTAGTGCAGCCAGGTCAATTGT-3′; EFNA1: S-5′-CATGAAGACCGCTGCTTGAG-3′, AS-5′-ATGTAGAACCCGCACCTCTG-3′; IL1b: S-5′-TGAGCTCGCCAGTGAAATGA-3′, AS-5′-AGATTCGTAGCTGGA-3′; RS9: S-5′-CGGAGACCCTTCGAGAAATCT-3′, AS-5′-GCCCATACTCGCCGATCA-3′; S1PR1: S-5′-CAGCAGCAAGATGCGAAGC-3′, AS-5′-CAGGGGTGGTTCGATGAGTG-3′; SOCS3: S-5′-CCATTCGGGAGTTCCTGGAC-3′, AS-5′-TTGGCTTCTTGTGCTTGTGC-3′; TGFbr1: S-5′-TCAAAAACTGGGTCTGTGACTACA-3′, AS-5′-ATCGACCTTTGCCAATGCTTTC-3′; TGFbr2: S-5′-GTCTATGACGAGCAGCGGG-3′, AS-5′-TCTGGGCCTCCATTTCCACA-3′. Quantification of mRNAs was normalized to the expression of RS9 as a reference gene using the Bio-Rad CFX Maestro 2.0 software v5.0.021.0616 (Bio-Rad Laboratories, Hercules, CA, USA).

### 2.6. Western Blotting

For protein expression analysis in microglia, microglia cells were treated with 10 µM S1P1 inhibitor NIBR0213 (21513, Cayman Chemical Company, Ann Arbor, MI, USA) or DMSO for 24 h. Cells were then washed with ice-cold PBS and lysed as previously described [18]. Proteins were separated on 4–12% Bis–Tris gels (Thermo Fisher Scientific) and transferred onto nitrocellulose membranes. The membranes were blocked at RT with 3% BSA in TBS–Tween for 1 h. Primary Abs against CH25H, JUNB, S1P1, and β-tubulin (loading control) were used (see Table S1). Horseradish peroxidase-conjugated goat anti-mouse or goat anti-rabbit (1:10,000, Jackson ImmunoResearch Laboratories, West Grove, PA, USA) were used as secondary Abs. The bands were visualized by chemiluminescence ECL reactions (Merck Millipore, Darmstadt, Germany), and band density was quantified by Quantity One^®^ software v4.6.6 (Bio-Rad Laboratories).

### 2.7. Flow Cytometry

Flow cytometry analysis was performed as previously described [20]. Specifically, cells were trypsinized and washed once with PBS. The cells were incubated with APC-conjugated CD16, CD32, CD86, CD150, CD163, and CD206 antibodies (Table S1) for 30 min at 4 °C in the dark, or alternatively with primary antibodies against PD-L1 (Table S1) for 45 min at 4 °C. For the detection of intracellular Iba-1 (Table S1), microglia cells were fixed with methanol for 20 min at −20 °C, then washed twice with 10% FCS-supplemented RPMI-1640. After washing with PBS, PDL-1 or Iba-1-stained cells were incubated with a secondary antibody for 45 min, at 4 °C in the dark. Cells were then washed with PBS and analyzed by flow cytometry. Antigen expression was analyzed by flow cytometer S1000EXi (Stratedigm, Inc., San Jose, CA, USA) with CellCapTure software v4.1 (https://stratedigm.com/cellcapture/ accessed on 17 January 2025) (Stratedigm, Inc.) and FlowJo v10 (FlowJo, Ashland, OR, USA). Dead cells were gated out from the analysis.

### 2.8. Construction of S1P1^lo^ Microglia Cells

Low-S1P1-expressing microglia cells (MG S1P1^lo^) were established by transduction with pGIPZ vector containing a shRNA sequence targeting S1P1 mRNA (RHS4430-200247965; Dharmacon, Lafayette, CO, USA). A sh-non-silencing pGIPZ vector (RHS4531, Dharmacon) was used to establish control microglia cells (MG S1P1^ctrl^). The production of viral vectors as well as microglia transduction were performed as previously described [18,19].

### 2.9. Viability Assay (XTT)

A total of 4 × 10^3^ microglia cells were seeded on a 96-well plate (3596, Corning Inc., Corning, NY, USA) for 24 h, then treated with 10 or 20 µM NIBR0213 or DMSO in starvation medium, or left untreated (in MG S1P1^ctrl^ and MG S1P1^lo^ cells), for an additional 24 h. Alternatively, 4 × 10^3^ microglia cells were seeded on a 96-well plate for 24 h, then pre-treated with 10 µM NIBR0213 or DMSO for 1 h, followed by treatment with MCM and either NIBR0213 or DMSO for 24 h. Cell viability was determined using Cell Proliferation Kit (XTT, 20-300-1000, Biological Industries, Kibbutz Beit Haemek, Israel). Briefly, the cells were incubated with XTT reagent prepared in growth medium according to the manufacturer’s instructions, for 1 h at 37 °C in the dark. The absorbance was then measured using Synergy H1 microplate reader (BioTek Instruments, Inc., Winooski, VT, USA) at a wavelength of 450 nm, with a reference wavelength of 630 nm to correct for background absorbance.

### 2.10. Phagocytosis Assay

A total of 4 × 10^4^ microglia cells were seeded in triplicates on a 24-well plate for 24 h, then treated with 10 µM NIBR0213 or DMSO in starvation medium for 24 h. An amount of 2 µL of opsonized beads were added to each well for 4 h. The cells were washed three times with PBS, trypsinized and centrifuged, and then fixed with cold methanol for 20 min. Following two washes with RPMI-1640 supplemented with 10% FCS, bead uptake was measured by flow cytometry as described above. Fluorescent latex beads (L1030, Sigma-Aldrich) were opsonized with RPMI-1640 supplemented with 10% FCS (1:10) for 1 h at 37 °C.

### 2.11. Apoptosis Assay

A total of 4 × 10^4^ microglia cells were seeded on a 24-well plate for 24 h, then treated with 10 or 20 µM NIBR0213 or DMSO in starvation medium for 6 h. Alternatively, 4 × 10^4^ microglia cells were seeded on a 24-well plate for 24 h, then pre-treated with 10 µM NIBR0213 or DMSO in starvation medium for 1 h, after which they were treated with MCM and NIBR0213 or DMSO for additional 6 h. Apoptotic and necrotic cells were quantified using Annexin V-FITC, which binds phosphatidylserine (PS), and propidium iodide (PI) staining with the MEBCYTO^®^ Apoptosis Kit (4700, MBL International, Woburn, MA, USA). Briefly, the cells were pelleted and washed once with PBS. Then, they were resuspended in binding buffer, to which Annexin V-FITC and PI were added, and incubated for 15 min at room temperature in the dark. Binding buffer was added to the cells again, and apoptosis rates were determined by flow cytometry as described above. Dead cells were not gated out in this experiment.

### 2.12. Cell Death Labeling in Co-Cultures

A total of 0.37 × 10^5^ mCherry-labeled BMMCs and 4 × 10^5^ microglia cells were co-cultured in 6cm plates for 24 h, then treated with 10 µM NIBR0213 or DMSO in starvation medium for 24 h. Cells were trypsinized, washed once with PBS, and DAPI (2879038, BioGems, Westlake Village, CA, USA) was added to the cells prior to reading in the flow cytometer.

### 2.13. Proliferation in Co-Cultures

A total of 1 × 10^3^ mCherry-labeled BMMCs and 1.1 × 10^3^ microglia cells were co-cultured in 96-well plates (3596, Corning Inc.) for 24 h. Alternatively, 2 × 10^3^ mCherry-labeled BMMCs or 2 × 10^3^ microglia cells were seeded separately in a 96-well plate. Cells were then treated with 10 µM NIBR0213 or DMSO in starvation medium. Images were captured every 3 h over a 72 h period using the IncuCyte S3 live-cell imaging system (Essen BioScience, Inc., Ann Arbor, MI, USA). Proliferation was quantified based on changes in cell-covered area (µm^2^).

### 2.14. Spheroid (3D) Cultures

A total of 1 × 10^3^ mCherry-labeled BMMCs and 1.1 × 10^3^ microglia cells in starvation medium, pre-treated with 10 µM NIBR0213 or DMSO, were plated onto 96-well low-attachment u-shaped plates (Greiner Bio-One, Frickenhausen, Germany). Alternatively, 1 × 10^3^ mCherry-labeled BMMCs and 1.1 × 10^3^ MG S1P1^ctrl^ or MG S1P1^lo^ in starvation medium were plated onto the above-mentioned plate. The plates were centrifuged for 5 min at 1000 rpm, and wells were imaged every 6 h over a 72 h period. Spheroid size was measured using the IncuCyte system (Essen BioScience, Inc.).

### 2.15. Evaluation of Sensitivity to Combined BRAF and S1P1 Inhibition

A total of 5 × 10^3^ BMMCs were seeded on a 96-well plate (3596, Corning Inc.) for 24 h. Then, the cells were treated with 5 µM Vemurafenib (PLX-4032, Selleck Chemicals, Houston, TX, USA), 20 µM NIBR0213, or both in starvation medium for 24 h. The viability of the remaining cells was measured by CCK-8 reagent (96992, Sigma-Aldrich). Briefly, the cells were incubated with CCK-8 reagent in growth medium according to the manufacturer’s instructions for 1 h at 37 °C in the dark. The absorbance was then measured using Synergy H1 microplate reader (BioTek Instruments, Inc.) at a wavelength of 450 nm, with a reference wavelength of 630 nm to correct for background absorbance.

### 2.16. Biostatistic Analysis

All experiments were conducted with a minimum of three independent biological replicates. All viability assays were performed in at least three technical replicates for each of these independent biological experiments. Data were analyzed using Student’s *t*-test and considered significant at *p*-values < 0.05. Bar graphs represent mean and standard error of the mean (SEM) across multiple independent experimental repeats.

## 3. Results

### 3.1. Brain-Metastasizing Melanoma Cells Upregulate S1PR1 in Microglia via IL-6

In efforts to explore the melanoma–microglia crosstalk, we analyzed gene expression alterations of microglia cells exposed to factors secreted by different BMMCs previously generated in our lab [21].

**Figure 1:**
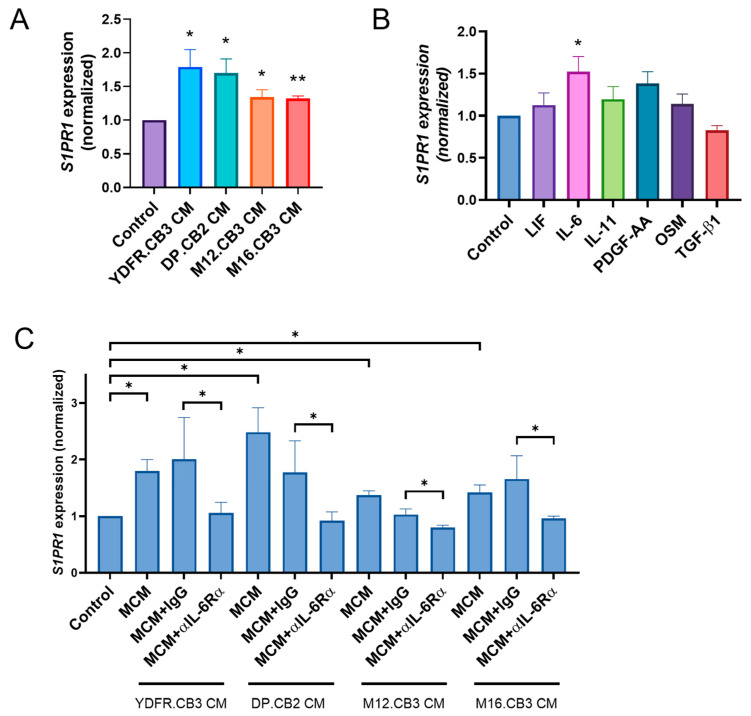
(**A**–**C**) Relative *S1PR1* mRNA expression in microglia treated for 3 h with (**A**) melanoma-conditioned medium (MCM) from four BMMC lines; (**B**) recombinant cytokines (LIF, 25 ng/mL; IL-6, 20 ng/mL; IL-11, 50 ng/mL; PDGF-AA, 20 ng/mL; OSM, 50 ng/mL; TGF-β1, 10 ng/mL); or (**C**) MCM, MCM + IgG isotype control, or MCM + αIL-6Rα antibody, as measured by RT-qPCR. RS9 served as the reference gene for normalization. Bars represent mean fold change in *S1PR1* expression relative to control + SEM. * *p* < 0.05, ** *p* < 0.01.

RNA-seq analysis of microglia treated with melanoma-conditioned medium (MCM), derived from two BMMC lines, revealed significant upregulation of *S1PR1* expression following 3 h of treatment [21]. This finding was validated in the present study by qPCR analysis of microglia treated with MCM from four different melanoma patients. MCM of YDFR.CB3, DP.CB2, M12.CB3, and M16.CB3 cells upregulated *S1PR1* expression in microglia by a mean fold change (FC) of 1.8, 1.7, 1.3, and 1.3, respectively (Figure 1A).

In order to identify the BMMC-associated factors that are involved in the upregulation of microglial *S1PR1*, we examined several candidate BMMC-expressed cytokines that, according to previous studies, may be involved in this regulation [21,22,23,24].

Microglia were treated for 3 h with leukemia inhibitory factor (LIF), oncostatin M (OSM), IL-6, IL-11, platelet-derived growth factor AA (PDGF-AA), and transforming growth factor-β1 (TGF-β1). qPCR analysis showed that IL-6 was the only cytokine to upregulate *S1PR1* expression in microglia (FC = −1.52, *p* < 0.05) (Figure 1B).

In view of our previous results showing that IL-6 activates STAT3/SOCS3 pathway [22] and that microglia cells express the heterodimeric IL-6 receptor (IL-6Rα/gp130) [21,22], we aimed to test the involvement of the specific IL-6Rα subunit on *S1PR1* expression in microglia with neutralizing antibodies. qPCR analysis showed that IL-6Rα neutralization abrogated *S1PR1* upregulation in microglia treated with MCM of all four melanoma cell lines (Figure 1C). These results demonstrate that melanoma cells positively regulate the expression of *S1PR1* in microglia and that this upregulation is mediated by melanoma-derived IL-6, through the activation of IL-6Rα.

### 3.2. Pharmacological and Genetic Inhibition of S1P1

The upregulation of *S1PR1* in melanoma-associated microglia indicates that this receptor or its interaction with the S1P ligand plays a role in melanoma–microglia crosstalk. To investigate this possibility, in the next set of experiments we employed the selective S1P1 antagonist NIBR0213 [25] to inhibit S1P1 function in microglia cells.

**Figure 2:**
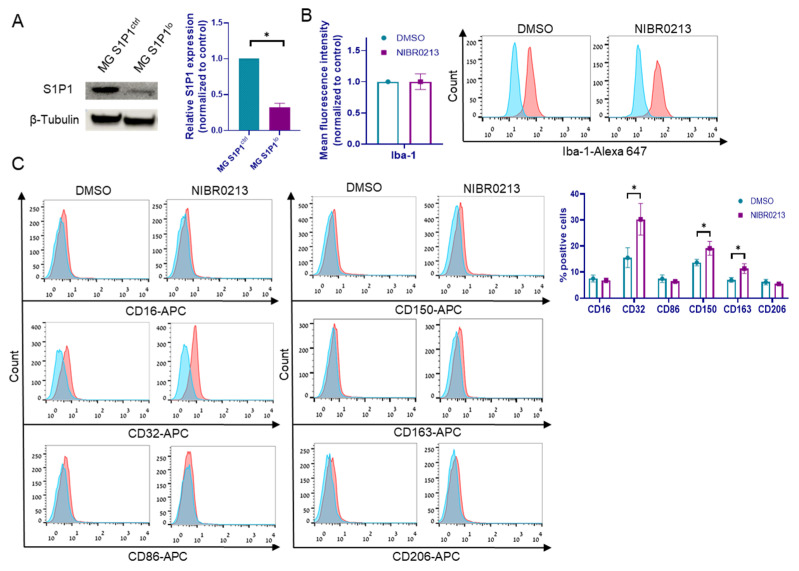

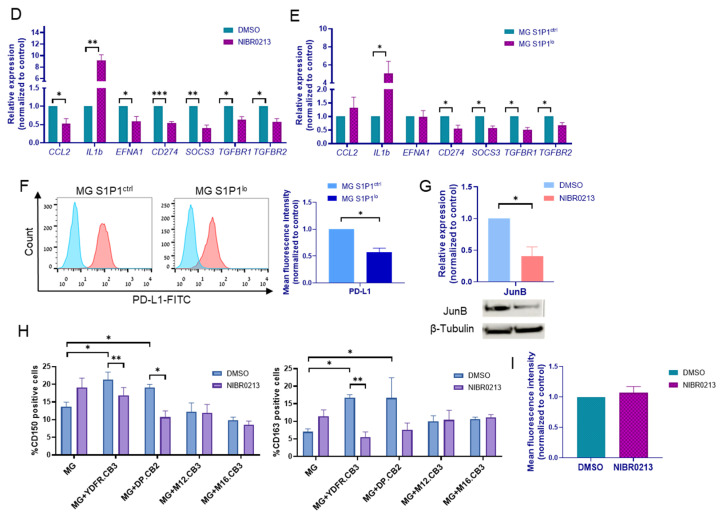
The effects of S1P1 inhibition on microglia. (**A**) Western blot analysis of S1P1 in S1P1^ctrl^ and S1P1^lo^ microglia. β-tubulin served for loading control. Representative blot of S1P1 expression is shown. Bars represent the mean S1P1 expression + SEM. * *p* < 0.05. (**B**) Iba-1 expression in NIBR0213- or DMSO-treated microglia, determined by flow cytometry. Representative histograms are shown. Bars represent the mean fluorescence intensity + SEM. (**C**) Expression of CD16, CD32, CD86, CD150, CD163, and CD206 in microglia treated with NIBR0213 or DMSO, determined by flow cytometry. Bars represent mean percentage of positive cells + SEM. * *p* < 0.05. Representative histograms are shown. (**D**,**E**) Relative expression of *CCL2*, *IL1B*, *EFNA1*, *CD274*, *SOCS3*, *TGFBR1*, and *TGFBR2* mRNA in microglia treated with NIBR0213 or DMSO (**D**), or in S1P1^ctrl^ and S1P1^lo^ microglia (**E**), detected by RT-qPCR. *RS9* served as the reference gene. Bars represent mean expression normalized to control + SEM. * *p* < 0.05, ** *p* < 0.01, *** *p* < 0.001. (**F**) PD-L1 expression in S1P1^ctrl^ and S1P1^lo^ microglia, determined by flow cytometry. Representative histograms are shown. Bars represent mean fluorescence intensity + SEM. * *p* < 0.05. (**G**) Western blot analysis of JunB in NIBR0213- or DMSO-treated microglia. β-tubulin served for loading control. Representative blot of JunB expression is shown. Bars represent the mean JunB expression + SEM. * *p* < 0.05. (**H**) CD150 and CD163 expression in microglia treated with NIBR0213 or DMSO, alone or co-cultured with BMMC, determined by flow cytometry. Bars represent the mean percentage of positive cells + SEM. * *p* < 0.05, ** *p* < 0.01. (**I**) Phagocytosis of fluorescent beads, determined by flow cytometry. Microglia were pre-treated with NIBR0213 or DMSO for 24 h, followed by 3 h incubation with fluorescent latex beads. Bars represent mean fluorescence intensity normalized to control + SEM. Original western blots are presented in File S1.

In addition, we generated microglia cells expressing low levels of S1P1 (S1P1^lo^) by lentiviral infection with shS1P1 constructs or with mock shRNA plasmids as control (S1P1^ctrl^). S1P1 downregulation was validated using Western blot analysis (Figure 2A).

These cells served, in some experiments, to validate the results obtained with the S1P1 antagonist. Interestingly, an attempt to generate S1P1 over-expressing microglia cells with lentiviral constructs resulted in extensive microglial cell death.

### 3.3. S1P1 Inhibition Shapes the Molecular Profile of Microglia Cells

Myeloid S1P1 is an important player in neuroinflammation [15] capable of mediating both anti or pro-inflammatory effects [7,8]. As such, S1P1 inhibition affects the inflammatory phenotype of myeloid cells [8].

In view of the findings that S1P1 is upregulated in BMMC-associated microglia, we characterized the effects of the NIBR0213 inhibitor on the activation state of such microglia. This was done by analyzing the expression of the pro-inflammatory markers CD16, CD32, CD86, and Iba-1, and of the anti-inflammatory markers CD150, CD163, and CD206. A 2 h treatment of microglia with NIBR0213 did not alter the expression of Iba-1, CD16, CD86, or CD206 (Figure 2B,C; Figure S1).

The treatment increased the expression of pro-inflammatory marker CD32 (from 15.5% to 30.2%) and of the anti-inflammatory markers CD150 and CD163 (from 13.6% to 19.1% and from 6.9% to 11.3%, respectively) (Figure 2B).

SOCS3 and PD-L1 suppress immune responses in macrophages and myeloid cells [26,27,28], and TGF-β signaling promotes the alternative polarization of tumor-associated macrophages [29]. In contrast, IL-1β is usually secreted by anti-tumor “M1” microglia, which play a key role in killing cancer cells. CCL2 recruits microglia and supports tumor cell migration and invasion [30,31,32], and EFNA1 is implicated in tumor cell migration, angiogenesis, and immune modulation [33,34].

Gene expression analysis of NIBR0213-treated microglia revealed upregulation of *IL1B* and downregulated expression of *CCL2*, *EFNA1*, *SOCS3*, and of the PD-L1-encoding *CD274*, as well as of TGF-β receptor type I (*TGFBR1*) and *TGFBR2* genes (Figure 2D). Similar results were obtained with MG S1P1^lo^ compared to MG S1P1^ctrl^ cells (Figure 2E), with the exception of *CCL2* and *EFNA1*, which remained unchanged following S1P1 knockdown.

In conformity with the gene expression data, FACS analysis indicated that the expression of PD-L1 protein was downregulated in MG S1P1^lo^ cells (Figure 2F). We thus conclude that S1P1 positively regulates PD-L1 expression.

We previously reported that the transcription factor JunB expressed by microglia induces an immunosuppressive phenotype in these cells and drives the progression of brain-metastasizing melanoma cells [21]. Here we found that inhibition of S1P1 by NIBR0213 downregulates JunB expression (Figure 2G).

The results summarized above suggest that inhibiting S1P1 in microglia shifts the overall balance of their responses toward an anti-tumor phenotype.

### 3.4. S1P1 Inhibition Abolishes CD150 and CD163 Upregulation in Microglia Co-Cultured with BMMC

In this set of experiments, we tested the effect of S1P1 inhibition on the expression level of the anti-inflammatory markers CD150 and CD163 in the context of melanoma–microglia interaction.

Microglia cells were cultured either alone or in co-culture with mCherry-labeled BMMC, then treated with NIBR0213 or DMSO as control for 24 h. The expression of CD150 and CD163 was higher in DMSO-treated (control) microglia co-cultured with YDFR.CB3 and DP.CB2 than in DMSO-treated microglia cultured alone, while expression remained unchanged in microglia co-cultured with M12.CB3 and M16.CB3. In microglia co-cultured with YDFR.CB3 and DP.CB2, NIBR0213 treatment decreased CD150 expression compared to DMSO treatment. However, it did not affect the expression of CD150 in microglia co-cultured with M12.CB3 and M16.CB3. Similarly, in microglia co-cultured with YDFR.CB3, NIBR0213 treatment decreased CD163 expression compared to DMSO treatment. However, it did not affect the expression of CD163 in microglia co-cultured with the other three cell lines (Figure 2H, Table A1).

The above results suggest an antagonistic interaction between microglial activation by melanoma (in some cases) and blocking S1P1. Their combined effect led to downregulation of CD150 (in the context of both YDFR.CB3 and DP.CB2) and of CD163 (in the context of YDFR.CB3), even though each factor alone induced protein upregulation. This suggests potential cross-inhibitory signaling and a context-dependent role of S1P1 signaling in regulating CD150 and CD163 expression in microglia.

It should be noted that inter-melanoma heterogeneity [22] is also evident in the current experiments. The impact of the four BMMC lines on microglial activation upon co-culture was not consistent across all four melanoma cell lines used in this study.

These results highlight a complex interplay between tumor-derived signals and intrinsic microglial pathways, where S1P1 acts as a critical modulator depending on the microenvironment.

### 3.5. S1P1 Inhibition Does Not Affect the Phagocytic Ability of Microglia Cells

S1P signaling is involved in all stages of the phagocytic process [35]. Given that phagocytosis is considered a pro-inflammatory function [36], and in light of the results presented above, it is reasonable to expect that S1P1 inhibition would not impair microglial phagocytic activity.

To test this, microglia were pre-treated for 24 h with either NIBR0213 or DMSO (control), followed by incubation with fluorescent latex beads together with NIBR0213 or DMSO for an additional 3 h. Phagocytosis was evaluated by flow cytometry, assessing bead uptake. Since no difference in mean fluorescence intensity was observed between the NIBR0213- and DMSO-treated cells (Figure 2I), we conclude that S1P1 inhibition does not affect the pro-inflammatory phagocytic function of microglia.

### 3.6. S1P1 Inhibition Reduces the Proliferation Rate of Microglia and BMMCs

S1P1 promotes the proliferation of many cell types including endothelial cells [37], esophageal squamous cell carcinoma cells [38], and microglia cells [39]. Here we tested the effect of the selective S1P1 antagonist NIBR0213 on the proliferation rate of microglia and of their interaction partners—BMMCs.

**Figure 3:**
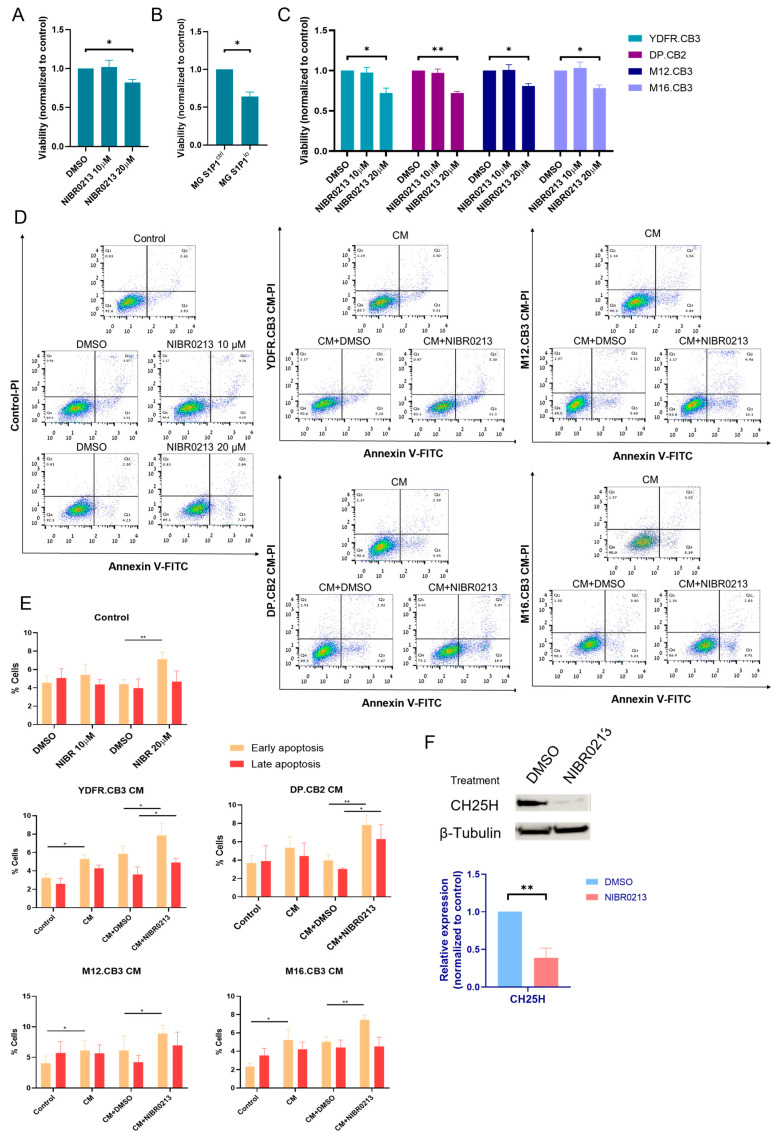
S1P1 regulates the viability of microglia cells and BMMCs. (**A**,**B**) Viability of microglia cells treated with NIBR0213 or DMSO for 24 h (**A**) or of S1P1^ctrl^ and S1P1^lo^ microglia cultured for 24 h (**B**) was measured by XTT. Bars show mean viability normalized to control + SEM. * *p* < 0.05. (**C**) Viability of BMMCs treated with NIBR0213 or DMSO for 24 h, measured by XTT. Bars show mean viability normalized to control + SEM. * *p* < 0.05, ** *p* < 0.01. (**D**,**E**) Early and late apoptosis of microglia cells exposed to BMMC-conditioned medium (CM) with NIBR0213 or DMSO, assessed by Annexin V/PI staining and flow cytometry. Control microglia cells were treated with NIBR0213 or DMSO in medium. (**D**) Representative flow cytometry dot plots are shown. (**E**) Bars show mean percentage of positive cells + SEM. * *p* < 0.05, ** *p* < 0.01. (**F**) Western blot analysis of CH25H in microglia treated with NIBR0213 or DMSO. β-tubulin served for loading control. Representative blot of CH25H is shown. Bars show mean CH25H expression + SEM. ** *p* < 0.01. Original western blots are presented in File S1.

Microglial cells were treated with 10 or 20 µM NIBR0213 for 24 h. Only the 20 µM concentration (but not 10 µM) significantly reduced microglial proliferation, as tested by XTT, compared to DMSO (control) (Figure 3A). Similarly, the proliferation of MG S1P1^lo^ was significantly lower than control microglia (MG S1P1^ctrl^) (Figure 3B).

We also assessed the effect of S1P1 inhibition on BMMCs. Similar to microglia, BMMCs treated with 20 µM NIBR0213 (and not 10 µM) displayed a reduced proliferation rate (Figure 3C).

These results suggest that S1P1 expression in microglia supports cell proliferation, and that S1P1 signaling is also involved in BMMC proliferation.

### 3.7. Melanoma-Secreted Factors Sensitize Microglia to S1P1 Inhibition-Induced Apoptosis

Subsequent to our previous findings that melanoma cells interact reciprocally, resulting, among other things, in an increase in microglial viability [16], we aimed to test the cytotoxic effect of S1P1 inhibition in melanoma-exposed microglia compared to the effect of this inhibition in naïve microglia. Propidium iodide (PI) and Annexin V staining showed that 10 µM NIBR0213 did not trigger apoptosis in naïve microglia, and only treatment with the higher concentration of 20 µM induced apoptosis.

However, in microglia exposed to melanoma CM, S1P1 inhibition with 10 µM NIBR0213 reduced cell viability compared to DMSO (control). The reduced viability was associated with an increased apoptotic cell death, suggesting that S1P1 inhibition by 10 µM NIBR0213 induces apoptosis in activated microglia exposed to melanoma-derived factors (Figure 3D,E).

S1P1 is known to promote cell survival [40] and its neutralization by a functional antagonist induced apoptosis in microglia. This effect was not mediated by the S1P ligand binding inhibition, but rather through activation of sterol regulatory element-binding protein 2 (SREBP2). In turn, SREBP2 was found to enhance the cleavage of poly-ADP-ribose-polymerase (PARP) and caspase-3 [41]. In addition, the enzymatic product of cholesterol 25-hydroxylase (CH25H), 25-hydroxycholesterol (25-HC), has been reported to suppress SREBP activation [42].

Based on these findings, we investigated whether S1P1 regulates CH25H, being an upstream modulator of SREBPs. Western blot analysis showed that treatment with NIBR0213 led to a reduction in CH25H expression in microglia (Figure 3F). These results suggest that S1P1 inhibition may suppress CH25H expression, potentially enhancing SREBP2 activity and thereby promoting apoptosis in microglial cells.

### 3.8. BMMCs Exhibit Greater Sensitivity to NIBR0213-Induced Growth Arrest Compared to Their Microglial Co-Culture Counterparts

**Figure 4:**
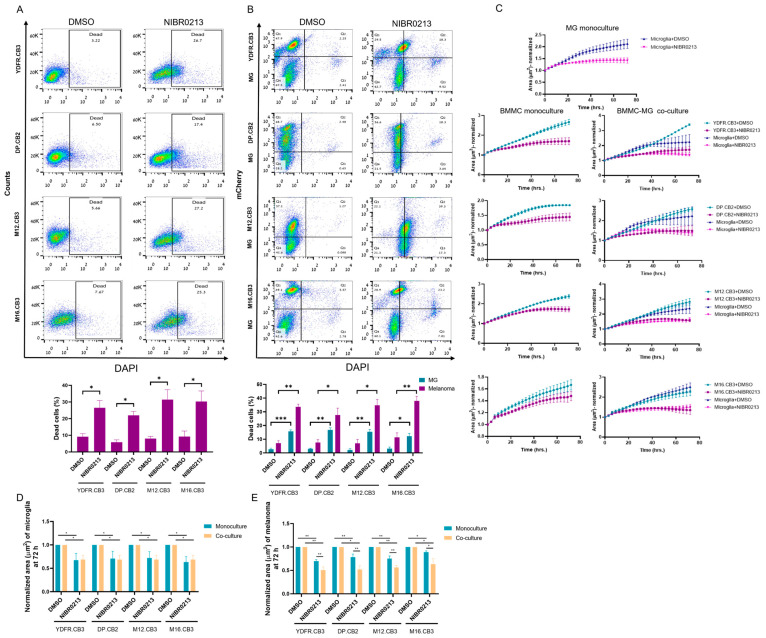
BMMCs show greater sensitivity to NIBR0213-induced cell death and growth arrest than microglia in melanoma–microglia co-cultures. (**A**,**B**) Cell death of BMMCs cultured alone (**A**), or BMMCs and microglia in co-cultures (**B**), assessed by DAPI staining and flow cytometry following treatment with NIBR0213 or DMSO. Representative flow cytometry dot plots are shown. Bars represent mean percentage of dead cells + SEM. * *p* < 0.05, ** *p* < 0.01.*** *p* < 0.001. (**C**) Microglia and mCherry-labeled BMMCs were co-cultured, or grown in monocultures, then treated with NIBR0213 or DMSO for 72 h. Images were acquired every 3 h using the Incucyte system, and cell area (µm^2^) was quantified at each time point. The microglia growth area in co-cultures was calculated by subtracting BMMC area from the total cell area. Data are shown as normalized mean area ± SEM. (**D**,**E**) End-point analysis (at 72 h) of (**C**) showing the mean area of NIBR0213-treated BMMC (**D**) or microglia (**E**), normalized to DMSO-treated controls in both monocultures and co-cultures. Bars represent mean + SEM * *p* < 0.05, ** *p* < 0.01.

Published work indicated that S1P1 inhibition induced apoptosis of murine melanoma cells [14]. Asking if S1P1 specific inhibition acts similarly on human BMMCs, we measured the effect of 20 µM NIBR0213 on the viability of BMMCs following a 24 h incubation. DAPI staining indicated cell death of all four BMMCs (Figure 4A).

As a tumor-promoting factor, S1P1 enhances tumor proliferation, migration, invasion, and neovascularization in various types of cancer [43,44]. In light of the observation that NIBR0213 induces cell death in BMMC and in melanoma-exposed microglia, we measured the effect of S1P1 inhibition on the viability of both BMMC and microglia cells in a co-culture setting, where these two cells interact.

Co-cultures composed of mCherry-labeled BMMCs and microglia cells were treated for 24 h with 10 µM NIBR0213, or DMSO as control. DAPI staining and flow cytometry analysis were employed to determine cell death. The results (Figure 4B, Table A2) revealed that all four BMMC lines, as well as microglia cells, were sensitive to NIBR0213-mediated cytotoxicity.

We next examined how S1P1 inhibition influences the proliferation rates of BMMCs and microglia over time in co-cultures. mCherry-labeled BMMCs were co-cultured with microglia cells and treated with either NIBR0213 or DMSO (control) for 3 days. Monocultures of these cells were cultured and treated similarly, for comparison. Incucyte-based analysis revealed that NIBR0213 treatment significantly reduced the proliferation of both microglia and BMMCs. While microglia showed a comparable reduction in proliferation when cultured alone or with BMMCs (Figure 4C,D, Table A3), the proliferation of BMMCs was more strongly inhibited by NIBR0213 in co-cultures than when cultured alone (Figure 4C,E, Table A4).

Overall, these findings indicate that BMMCs are more susceptible than microglia to NIBR0213-induced growth inhibition, particularly when in direct interaction with microglia.

### 3.9. S1P1 Inhibition Modulates Spheroid Formation in BMMC–Microglia Co-Cultures

3D cultures better replicate the in vivo cell–cell interaction and architecture [45]. To investigate the role of S1P1 in BMMC–microglia interactions, we used 3D co-cultures of mCherry-labeled BMMCs with microglia, treated with either NIBR0213 or DMSO for 72 h. We then assessed the ability of the cells to form spheroids.

**Figure 5:**
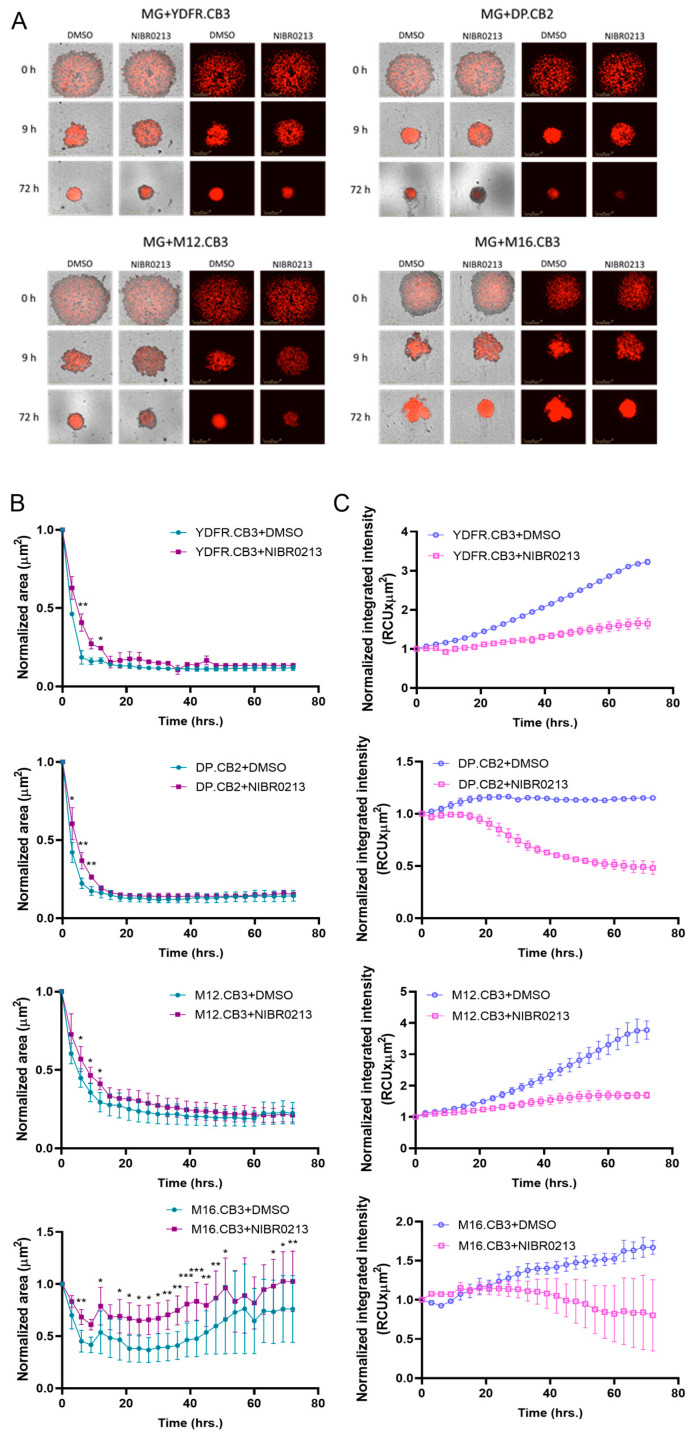

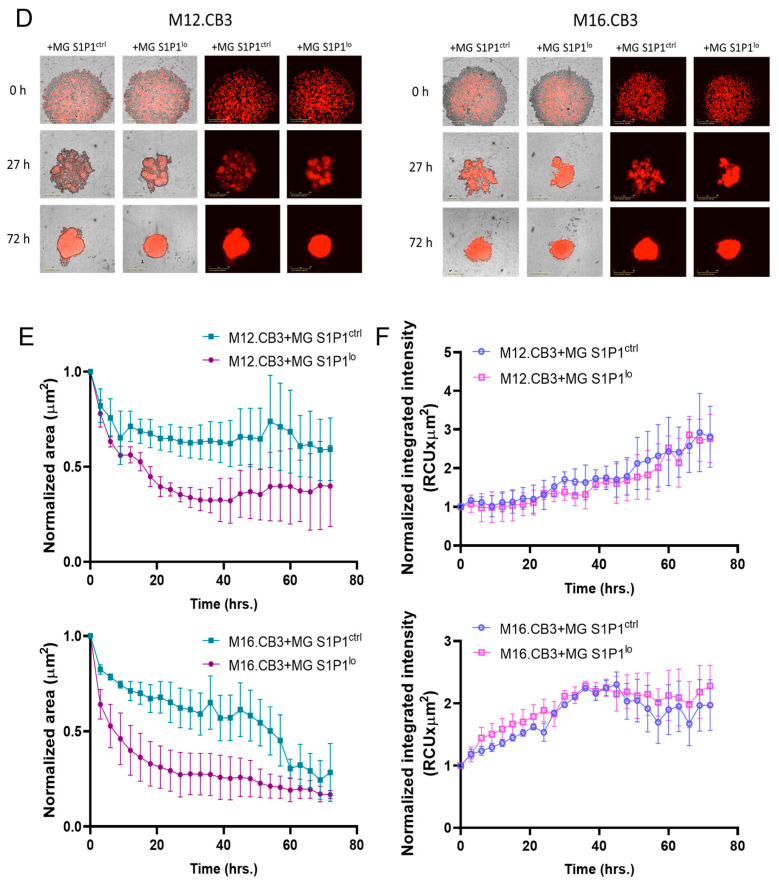
S1P1 regulates spheroid formation in BMMC–microglia 3D co-cultures. (**A**–**C**) Spheroid formation assay of microglia and mCherry-labeled BMMCs treated with NIBR0213 or DMSO. Cultures were imaged every 3 h for 72 h using the Incucyte system. Representative images at 0, 9, and 72 h are shown (**A**). Graphs show mean normalized spheroid area (µm^2^) ± SEM (**B**) and mean normalized mCherry integrated intensity (RCUxµm^2^) ± SEM. * *p* < 0.05, ** *p* < 0.01.*** *p* < 0.001. (**C**). Scale bar: 400 μm. (**D**–**F**) Spheroid formation assay of mCherry-labeled BMMCs and S1P1^ctrl^ or S1P1^lo^ microglia. Cultures were imaged every 3 h for 72 h using the Incucyte system. Representative images of the wells at 0, 27, and 72 h are shown (**D**). Graphs show mean normalized spheroid area (µm^2^) ± SEM (**E**) and mean normalized mCherry integrated intensity (RCUxµm^2^) ± SEM (**F**). Scale bar: 400 μm.

During the initial phase of spheroid aggregation, in the first 9 h of the experiment, we observed a higher shrinkage rate in the control spheroids treated with DMSO than in the NIBR0213-treated spheroids, suggesting that NIBR0213 inhibits the ability of tumor cells to form aggregates, and indicating a more aggressive phenotype of the DMSO-treated ones (Figure 5A,B), probably due to higher motility [46]. In YDFR.CB3 and M12.CB3 co-cultures, this difference persisted for an additional 3 h, and in M16.CB3 co-cultures, this difference persisted for an additional 40 h. Thereafter and over time, spheroid areas became comparable between NIBR0213-treated vs. DMSO-treated spheroids.

To estimate BMMC content within the spheroids, mCherry integrated fluorescence intensity was measured. Across all four BMMC lines, from around 20 h of treatment, mCherry intensity was significantly higher in DMSO-treated spheroids than in NIBR0213-treated spheroids (Figure 5A,C), suggesting a higher proliferation rate in the DMSO-treated spheroids, either due to an increased proliferative ability, or increased cell growth arrest/death of the NIBR0213-treated BMMCs.

As tumor cell spheroid formation rate accounts as a measure for its aggressiveness [45,46], we conclude that S1P1 inhibition reduces the malignancy phenotype of melanoma cells, perhaps by inhibiting the tumor-supportive [16] melanoma–microglia interactions.

We next examined whether genetic suppression of S1P1 in microglia affects spheroid formation in 3D co-cultures. To this end, we co-cultured the four mCherry-labeled BMMCs with microglia expressing low levels of S1P1 (MG S1P1^lo^) and compared their aggregation capacity to that of control microglia (MG S1P1^ctrl^)–BMMC co-cultures. This setup allowed us to specifically assess the impact of microglial S1P1 knockdown on spheroid formation. In this experiment, 3D co-cultures containing S1P1^lo^ microglia formed smaller, more compact spheroid structures compared to those formed with control microglia (MG S1P1^ctrl^) in the presence of M12.CB3 and M16.CB3 cells (Figure 5D,E). In contrast, spheroid area remained unchanged in co-cultures with YDFR.CB3 and DP.CB2.

Despite the smaller size, the integrated mCherry intensity in S1P1^lo^ microglia–BMMC spheroids was comparable to that of S1P1^ctrl^ microglia–BMMC co-cultures (Figure 5D,F), indicating that the reduced spheroid area was not due to a lower number of BMMCs.

The results demonstrate that S1P1 positively regulates cell aggregation and BMMC proliferation in microglia–BMMC 3D co-cultures. Whereas pharmacological inhibition of S1P1 (via NIBR0213) delays spheroid formation and reduces melanoma cell proliferation across all tested BMMC lines, genetic downregulation of S1P1 in microglia results in smaller, denser spheroids with certain BMMC lines (M12.CB3 and M16.CB3), though not all. Importantly, the reduction in spheroid size in these two BMMC lines was not due to fewer melanoma cells, suggesting that S1P1 primarily influences the structural organization and aggregation dynamics of the co-cultures.

These results suggest that the proliferation of BMMCs in BMMC–microglia co-cultures is regulated, at least in part, by S1P–S1P1 interactions.

These findings accentuate both the functional relevance of microglial S1P1 in tumor–stroma interactions and the need to consider varied responses of melanoma tumors from different patients to different types of stimuli [47,48].

### 3.10. Targeting S1P1 Potentiates the Efficacy of Vemurafenib Treatment

Previous studies have suggested that targeting the S1P/S1P1 signaling axis could be beneficial in treating certain cancer types [49,50]. In this pilot experiment, we explored whether S1P1 inhibition could be employed in melanoma treatment. The BRAFV600E mutation is the most prevalent BRAF alteration in melanoma [51], and the selective BRAFV600E inhibitor, Vemurafenib, is FDA-approved for treatment of metastatic melanoma [52]. Non-FDA-approved indication of Vemurafenib also includes metastatic and unresectable melanoma with BRAF V600K mutation [53].

The BMMC lines used in this study carry BRAFV600 mutations: YDFR.CB3, DP.CB2, and M16.CB3 harbor BRAFV600E, while M12.CB3 carries the BRAFV600K variant (Dave S. Hoon, personal communication). To determine whether S1P1 inhibition could enhance the cytotoxicity of Vemurafenib, we treated BMMCs with 5 µM Vemurafenib, 20 µM NIBR0213, or a combination of both, and assessed cell viability after 24 h.

**Figure 6:**
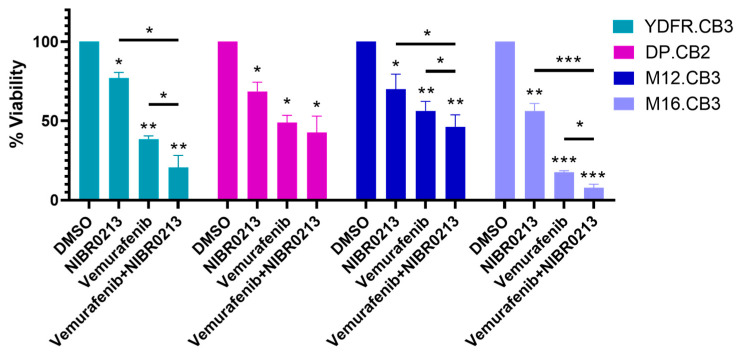
S1P1 inhibition enhances therapeutic efficacy of Vemurafenib. Viability of BMMCs treated for 24 h with NIBR0213, Vemurafenib, Vemurafenib + NIBR0213, or DMSO (control) was measured using the CCK-8 assay. Bars represent the mean percentage of viable cells normalized to DMSO + SEM. Asterisks indicate statistical significance compared to DMSO. * *p* < 0.05, ** *p* < 0.01, *** *p* < 0.001.

The results (Figure 6, Table A5) demonstrated that combined treatment with Vemurafenib and NIBR0213 reduced cell viability more than either agent alone in YDFR.CB3, M12.CB3, and M16.CB3 cultures (Figure 6, Table A5). In contrast, DP.CB2 cells did not show increased sensitivity to the combination therapy compared to monotreatment with each treatment alone.

## 4. Discussion

The interactions between cancer cells and non-cancerous cells in the tumor microenvironment are pivotal in the progression towards metastasis [54]. Such interactions may either prevent or drive metastatic progression, as cancer cells have the capacity to modulate anti-tumor functions of non-cancerous microenvironmental cells toward tumor supporting functions [4]. For example, tumor-associated, immunosuppressive, anti-inflammatory macrophages facilitate tumor growth and metastasis [55]. They acquire this property via contact with cancer cells [56,57].

Our lab identified and characterized molecules involved in the crosstalk between brain-metastasizing melanoma cells (BMMCs) and non-cancerous cells in the brain microenvironment, specifically microglia [21,22].

Given the inter-tumor heterogeneity previously described by our lab [22,47,48] as well as by others [58,59], we concentrated on molecules that behave similarly in the context of these interactions in melanoma cells from multiple melanoma patients. We observed that a short-term exposure to factors secreted from BMMCs derived from four different patients upregulated *S1PR1* gene expression in microglia cells. IL-6 released from the melanoma cells was identified as the factor that upregulated microglial *S1PR1* expression. Of note, the S1P1-IL-6 crosstalk is reciprocal—the S1P/S1P1 pathway regulates IL-6 expression, and vice versa [60], suggesting a positive feedback loop that may reinforce S1P1 signaling in the tumor microenvironment.

The S1P/S1P1 axis regulates cell survival, motility, and angiogenesis and its deregulation impacts cancer progression. The involvement of this axis in cancer progression may be related to alterations in the expression of S1P receptors [61]. The IL-6-mediated S1P1 upregulation observed in melanoma-associated microglia in this study may represent such a mechanism.

Here, we evaluated the role played by S1P1 in the regulation of the molecular profile and functions of microglia cells by monitoring the consequences of S1P1 inhibition on these cells either when grown alone in monocultures or in association with brain-metastasizing melanoma cells.

Consistent with previous reports of dual functions for S1P1 in inflammation [7,35], our results suggest that S1P1 signaling maintains an immunosuppressive phenotype in microglia. Inhibition of S1P1 shifted microglia toward a pro-inflammatory profile, as evidenced by increased CD32 expression and decreased expression of immunosuppressive regulators such as *SOCS3*, PD-L1, and TGF-β receptors, *TGFBR1* and *TGFBR2*. Similar findings were obtained in S1P1^lo^ microglia.

The results presented above indicate that inhibition of S1P1 shifted microglia toward a pro-inflammatory phenotype, as evidenced by increased CD32 expression, which can activate the NF-κB and MAPK pathway, resulting in the secretion of pro-inflammatory cytokines such as TNF-α, IL-1β, and IL-8 [62].

SOCS3 is a negative regulator of the STAT3 signaling pathway [63]. Its downregulation facilitates STAT3 activation [64,65], which in turn mediates inflammatory responses in microglia [66,67]. SOCS3-deficient macrophages express pro-inflammatory cytokines and prolong the survival of glioma-bearing mice [68]. Our previous work showed that SOCS3 overexpression in microglia cells enhanced melanoma malignancy [22].

CD150 and CD163 upregulation in melanoma-exposed microglia further suggests a tumor-driven immunosuppressive polarization [69,70]. This polarization appears to be mediated, at least in part, by S1P1 signaling, as upon S1P1 inhibition, CD150 expression is reduced in microglia co-cultured with two BMMC lines, and CD163 expression is reduced in those co-cultured with YDFR.CB3 cells. These findings demonstrate that S1P1 inhibition can counteract melanoma-induced upregulation of the anti-inflammatory markers CD150 and CD163 in microglia, but only in a subset of melanoma lines, highlighting both the context-dependent role of S1P1 signaling and the importance of inter-tumoral heterogeneity in shaping microglia responses. PD-L1 expression in microglia plays a key role in modulating immune responses within the central nervous system (CNS), including in the context of brain tumors such as glioblastoma or brain metastases. In mouse models of glioblastoma, PD-L1 knockout in microglia increased T cell recruitment and prolonged survival [71]. In human glioma samples, PD-L1 expression in microglia correlated with poor prognosis and lower infiltration of T cells into the tumor [72].

TGF-β, a key modulator of tumor-associated macrophages (TAMs), promotes their alternative (M2-like) activation [29], shifting them toward a phenotype that supports tumor cell proliferation and invasion, ultimately contributing to tumor immune evasion [73,74]. TGF-β signals via TGF-β receptors I and II, and reduction in receptor expression leads to a subsequent decrease in response to TGF-β [75]. The significant decrease of *TGFBR1* and *TGFBR2* in microglia following S1P1 inhibition by NIBR0213 (or knockdown), as reported above, further supports the conclusion that S1P1 functions as an immunosuppressive and pro-metastatic factor in melanoma brain metastasis.

RNA-seq analysis revealed that JunB was strongly upregulated in microglia exposed to melanoma-derived factors, particularly via LIF–JAK/STAT3 signaling. Microglia cells expressing relatively high levels of the transcription factor JunB enhanced melanoma malignancy by exerting anti-inflammatory and immunosuppressive functions. Low JunB microglia showed an anti-tumor activity [21], and since S1P1 inhibition reduced JunB expression, our findings suggest that S1P1 may contribute to tumor-promoting microglial functions by maintaining JunB expression.

Overall, gene expression analysis of microglia with inhibited S1P1 signaling pointed toward an immunostimulatory, pro-inflammatory phenotype resembling an “M1-like” state, potentially supporting recruitment of cytotoxic immune cells and tumor suppression.

S1P1 promotes proliferation of different types of cells [37,38,39]. We therefore tested the impact of S1P1 inhibition on cell growth in co-cultures of BMMCs and microglia, in an attempt to mimic the metastatic microenvironment of melanoma brain metastases. We also compared the inhibitory effect in cells when grown alone to the effect when grown in co-cultures.

In functional assays, S1P1 inhibition decreased proliferation and viability of both microglia and BMMCs, with melanoma cells being more sensitive than microglia. This may reflect melanoma’s reliance on pro-survival pathways, such as Mcl-1, which are influenced by S1P1 activity [40,76], as well as dysregulated sphingolipid metabolism in melanoma [11]. Furthermore, melanoma-conditioned microglia became more susceptible to apoptosis under S1P1 inhibition, suggesting that tumor-secreted factors sensitize microglia to S1P1 blockade.

As shown above, microglia cells may also secrete pro-apoptotic factors. This secretion may be promoted by NIBR0213 treatment upregulating *IL1B* expression, which, in turn, promotes the secretion of TNF-α [77]. This cytokine, in combination with S1P1 inhibition, would cause the reduction in the proliferation of melanoma cells co-cultured with microglia, compared to melanoma cultured alone.

The S1P/S1P1 axis mediates pro-survival signals and protects against apoptosis in many normal and malignant cell types exposed to pro-apoptotic stimuli [40].

Most interestingly, factors secreted by melanoma cells rendered microglia more susceptible to apoptosis triggered by S1P1 inhibition. The melanoma-derived pro-apoptotic factors may be either soluble or packed in extracellular vesicles. These results highlight a context-dependent function of S1P1 signaling in microglial survival. In resting microglia, S1P1 activity appears to be relatively low or tightly regulated, leading to minimal reliance on this pathway for maintaining viability. In contrast, exposure to melanoma-conditioned medium induces phenotypic shifts in microglia, potentially involving increased S1P1 expression and/or activation of survival pathways that facilitate adaptation to the tumor microenvironment.

This variation in sensitivity suggests the presence of heterogeneous microglial subsets within metastatic niches. Importantly, selectively targeting the S1P1-high subpopulation may offer a strategy to eliminate tumor-supportive, anti-inflammatory, and immunosuppressive microglia.

Mechanistically, our data are consistent with a model in which S1P1 may positively regulate CH25H in microglia. Inhibition of S1P1 decreased CH25H, which could relieve suppression of SREBP activity and thereby promote apoptosis [41,78,79]. This pathway may underlie the increased sensitivity of tumor-activated microglia to S1P1 inhibition.

Melanoma and microglia cells may interact either via secreted extracellular factors (vesicles or soluble factors) or by direct contact, for instance by ligand–receptor interaction, gap junctions, and tunnel nanotubes. The short distance between the cells in this case enables the passage of large molecules and even organelles like mitochondria to the neighboring cell, and hence encompasses broader possibilities for cells to affect their neighbors [80]. The influence of S1P1 and its inhibition on melanoma–microglia interaction was approached by measuring melanoma–microglia spheroid formation in the presence or absence of NIBR0213. This inhibitor caused a delayed aggregation of melanoma and microglia. We thus tentatively conclude that S1P1 inhibition disrupts melanoma–microglia cell communication.

In another set of experiments, we asked if BMMCs co-cultured with microglia whose S1P1 was knocked down (S1P1^lo^ microglia—see above) will also exhibit a delayed co-aggregation. Unexpectedly, this did not occur. M12.CB3 and M16.CB3 cells co-cultured with S1P1^lo^ microglia formed denser spheroids compared to co-cultures of melanoma and microglia that express S1P1. This divergence suggests that S1P1 inhibition in microglia alone is insufficient to disrupt melanoma–microglia aggregation and that the disruption of melanoma–microglia physical contact is dependent mostly on S1P1 inhibition in the melanoma partner. Taken together, we demonstrated that S1P1 assists in the establishment of a direct contact between BMMCs and microglia.

The S1P/S1P1 signaling axis has been proposed as a therapeutic target in various cancers [43,49]. Given the central role of S1P1 in modulating both microglia and melanoma cell responses, we explored its potential as a combinatory therapeutic target. We investigated whether combination therapy of S1P1 and BRAF inhibition could improve the efficacy of the widely used BRAF inhibitor (BRAFi), Vemurafenib. The rationale behind this therapeutic combination is that BRAFi-resistant melanoma cells exhibit elevated S1P1 expression [81], whereas BRAFi-sensitive cells express reduced levels of the receptor [82]. Our data corroborate these observations. The simultaneous treatment of BMMC lines with Vemurafenib and NIBR0213 led to an additive reduction in cell viability in three out of four cell lines, compared to either treatment alone.

In line with these observations, antagonizing S1P1 on melanoma-associated microglia reverses the immunosuppressive microenvironment induced by this receptor and promotes a pro-inflammatory shift in microglia. We hypothesize that this beneficial function is mediated by targeting anti-inflammatory microglia by the S1P1 inhibitor. The S1P1 inhibitor targets both melanoma cells as well as melanoma-activated microglia and induces apoptotic death in microglia. We suggest that CH25H located downstream to S1P1 is involved in NIBR0213-induced apoptosis of melanoma-activated microglia. Taken together, our findings highlight S1P1 as a potential therapeutic target in melanoma brain metastasis. Further studies are warranted to evaluate the efficacy of S1P1 inhibition, alone or in combination with BRAFi, in defined patient subpopulations, particularly those with limited response to BRAF-targeted therapies.

Taken together, our results suggest that S1P1 signaling contributes to immunosuppressive microglial polarization, melanoma–microglia interactions, and therapy resistance. While these findings provide evidence for a mechanistic role of S1P1 in shaping the tumor-supportive microenvironment, additional in vivo studies will be needed to establish direct causality in metastatic outgrowth.

## 5. Conclusions

Our findings suggest that S1P1 contributes to maintaining the immunosuppressive, tumor-promoting phenotype of melanoma-associated microglia in the brain metastatic niche. IL-6 secreted by brain-metastasizing melanoma cells upregulates S1P1 expression in microglia. Inhibition of S1P1 reduces the expression of immunosuppressive markers, decreases proliferation, and induces a shift toward a pro-inflammatory, anti-tumor phenotype. These phenotypic changes, along with impaired melanoma–microglia interactions, support the conclusion that S1P1 shapes a tumor-supportive microenvironment.

The S1P1 inhibitor targets both melanoma cells and melanoma-activated microglia and induces apoptotic death in microglia. Finally, the pilot experiments reported above support the potential of targeting the S1P–S1P1 axis to enhance the efficacy of BRAF inhibitor treatment and position the S1P/S1P1 axis as a promising therapeutic target in melanoma brain metastasis.

## Data Availability

The original contributions presented in this study are included in the article/Supplementary Material. Further inquiries can be directed to the corresponding author.

## Supplementary Materials

The following supporting information can be downloaded at https://www.mdpi.com/article/10.3390/cancers17193175/s1, Table S1: List of antibodies utilized in the study. Figure S1: Mean fluorescence intensity (MFI) of inflammation-associated markers. File S1: Original western blots.

## Abbreviations

The following abbreviations are used in this manuscript:

25-HC	25-hydroxycholesterol
BMMCs	Brain-metastasizing melanoma cells
CH25H	Cholesterol 25-hydroxylase
LIF	Leukemia inhibitory factor
MG	Microglia
OSM	Oncostatin M
PDGF-AA	Platelet-derived growth factor AA
S1P	Sphingosine-1-phosphate
S1PR1/S1P1	Sphingosine-1-phosphate receptor 1
SREBP2	Sterol regulatory element-binding protein 2
SphK1	Sphingosine-1-kinase
TAMs	Tumor-associated macrophages
TGF-β1	Transforming growth factor-β1
TGFBR1	TGF-β receptor type I
TGFBR2	TGF-β receptor type II

## Appendix A

### Appendix A.1

**Table A1:** CD150 and CD163 expression in microglia cells, alone or co-cultured with BMMC, treated with NIBR0213 or DMSO, was determined using flow cytometry. Data are presented as mean percentage of positive cells and SEM.

		CD150		CD163	
	Treatment	Average	SEM	Average	SEM
**MG**	DMSO	13.67	1.24	6.99	0.90
	NIBR0213	19.13	2.66	11.35	1.83
**MG+YDFR.CB3**	DMSO	21.30	2.14	16.73	0.87
	NIBR0213	16.90	2.16	5.55	1.43
**MG+DP.CB2**	DMSO	19.10	0.84	16.66	5.74
	NIBR0213	10.74	1.72	7.57	1.96
**MG+M12.CB3**	DMSO	12.19	2.54	10.01	1.61
	NIBR0213	11.93	2.38	10.39	2.76
**MG+M16.CB3**	DMSO	9.82	0.89	9.95	1.31
	NIBR0213	8.56	1.03	11.16	0.74

### Appendix A.2

**Table A2:** Cell death of microglia and BMMC in co-cultures as determined by DAPI staining using flow cytometry following treatment with NIBR0213 or DMSO. Data are presented as mean percentage of dead cells and SEM.

		MG		BMMC	
	Treatment	Average	SEM	Average	SEM
**MG+YDFR.CB3**	DMSO	2.73	0.78	7.18	1.71
	NIBR0213	15.78	1.08	33.59	2.06
**MG+DP.CB2**	DMSO	3.05	0.41	7.55	2.14
	NIBR0213	16.78	1.59	27.69	5.02
**MG+M12.CB3**	DMSO	2.05	1.05	6.98	2.90
	NIBR0213	15.43	1.80	34.83	4.17
**MG+M16.CB3**	DMSO	3.17	1.15	11.43	3.23
	NIBR0213	12.18	2.08	37.93	3.44

### Appendix A.3

**Table A3:** The proliferation rate of microglia alone or co-cultured with BMMC at 72 h was measured as the mean area of NIBR0213-treated microglia normalized to DMSO-treated microglia and SEM.

	Treatment	Average	SEM
**MG only**	DMSO	1.00	0.00
	NIBR0213	0.69	0.09
**MG+YDFR.CB3**	DMSO	1.00	0.00
	NIBR0213	0.67	0.14
**MG+DP.CB2**	DMSO	1.00	0.00
	NIBR0213	0.71	0.16
**MG+M12.CB3**	DMSO	1.00	0.00
	NIBR0213	0.72	0.14
**MG+M16.CB3**	DMSO	1.00	0.00
	NIBR0213	0.63	0.12

### Appendix A.4

**Table A4:** The proliferation rate of BMMC alone or co-cultured with microglia at 72 h was measured as the mean area of NIBR0213-treated BMMC normalized to DMSO-treated BMMC and SEM.

		Alone		Co-Culture	
	Treatment	Average	SEM	Average	SEM
**MG+YDFR.CB3**	DMSO	1.00	0.00	1.00	0.00
	NIBR0213	0.70	0.03	0.51	0.07
**MG+DP.CB2**	DMSO	1.00	0.00	1.00	0.00
	NIBR0213	0.78	0.07	0.52	0.08
**MG+M12.CB3**	DMSO	1.00	0.00	1.00	0.00
	NIBR0213	0.75	0.06	0.57	0.04
**MG+M16.CB3**	DMSO	1.00	0.00	1.00	0.00
	NIBR0213	0.89	0.03	0.63	0.12

### Appendix A.5

**Table A5:** Viability of BMMC treated with NIBR0213, Vemurafenib, Vemurafenib + NIBR0213, or DMSO for 24 h, as measured by XTT. Data are presented as the mean percentage of viable cells, normalized to DMSO, and SEM.

	YDFR.CB3		DP.CB2		M12.CB3		M16.CB3	
	Average	SEM	Average	SEM	Average	SEM	Average	SEM
DMSO	100	0	100	0	100	0	100	0
Vemurafenib	38.50	2.02	49.00	4.58	56.33	6.01	17.67	0.88
Vemurafenib+NIBR0213	20.50	7.79	42.67	10.33	46.33	7.51	7.83	2.24
NIBR0213	77.00	3.61	68.67	5.78	63.67	13.48	56.33	4.70
