# Supplementary material for: The S1P/S1P1 Signaling Axis Plays Regulatory Functions in the Crosstalk Between Brain-Metastasizing Melanoma Cells and Microglia
Source: Cancers (Basel). 2025 Sep 29;17(19):3175. doi: 10.3390/cancers17193175 (PMC12523612; doi:10.3390/cancers17193175)

Western blots- raw data

Figure 2A

30.10.23

MG con^{lo} MG S1P1^{lo} X X

S1P1

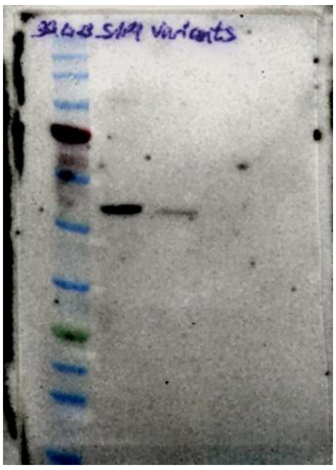

β-Tubulin

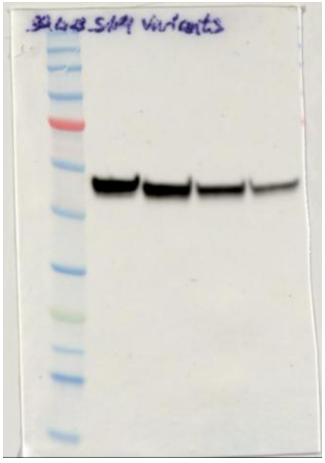

Protein ladder used- Cat no. PM007-0500, GeneDirex

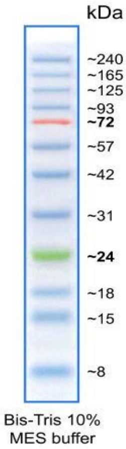

1.10.23

MG con^{lo} X X X X X MG S1P1^{lo} (clone) X

S1P1

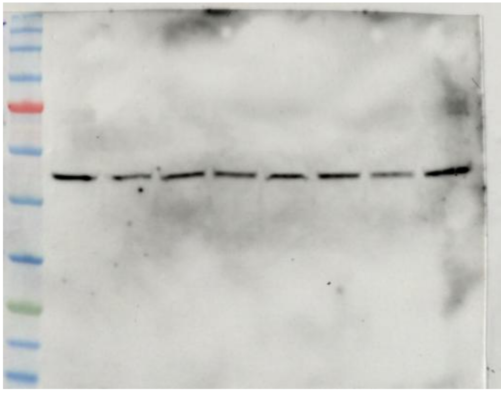

β-Tubulin

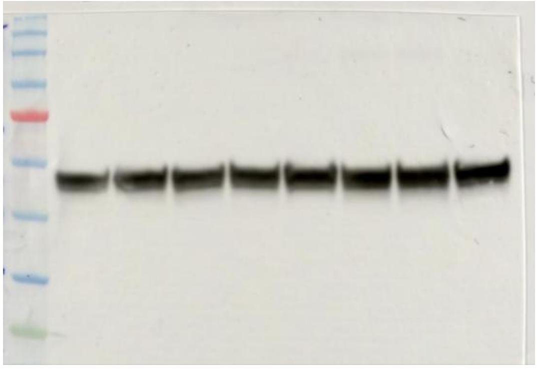

Western blots- raw data

Figure 2F, 3E

7.2.24

DMSO NIBR0213 DMSO NIBR0213

JunB

CH25H

β-Tubulin

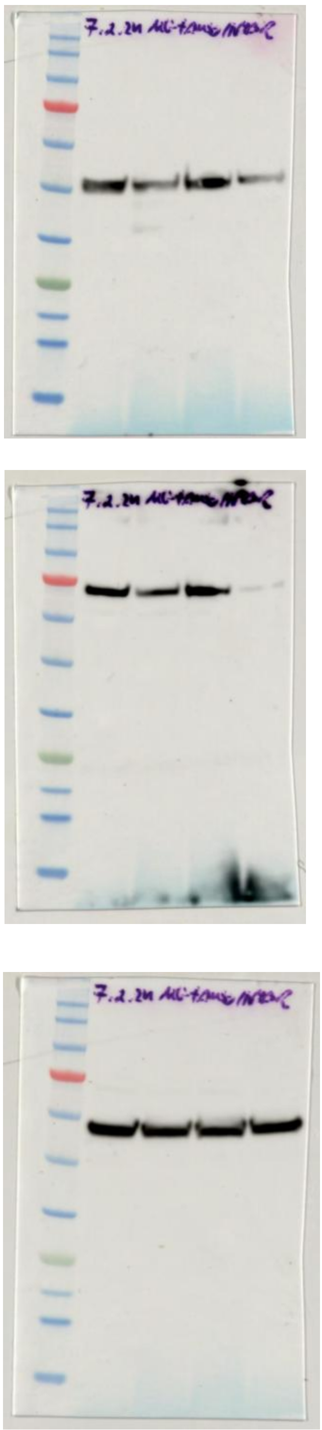

4.3.24

X X DMSO NIBR0213

JunB

CH25H

β-Tubulin

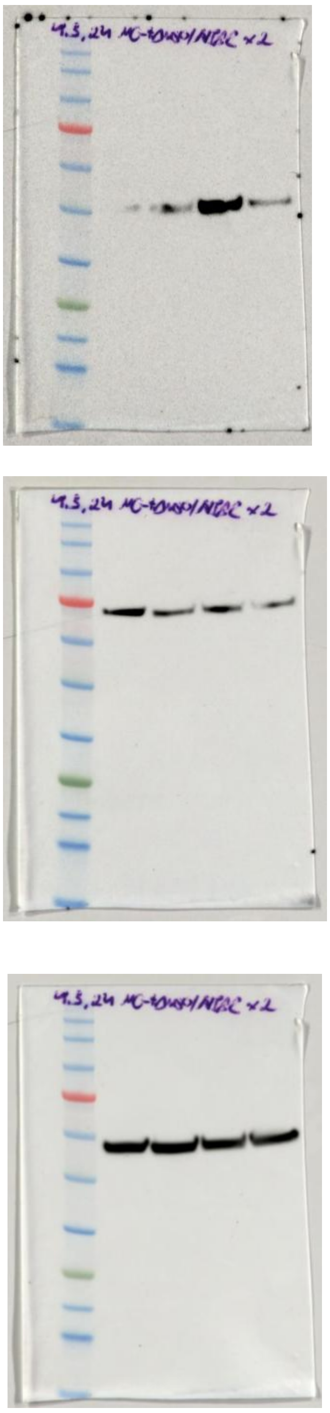

Supplement: Supplementary file 1 [file cancers-17-03175-s001.zip › cancers-3721559-file S1.pdf]
