# Supplementary material for: The S1P/S1P1 Signaling Axis Plays Regulatory Functions in the Crosstalk Between Brain-Metastasizing Melanoma Cells and Microglia
Source: Cancers (Basel). 2025 Sep 29;17(19):3175. doi: 10.3390/cancers17193175 (PMC12523612; doi:10.3390/cancers17193175)
Supplement: Supplementary file 1 [file cancers-17-03175-s001.zip › cancers-3721559-supplementary.pdf]

Supplementary Materials

The S1P/S1P1 Signaling Axis Plays Regulatory Functions in the Crosstalk Between Brain-Metastasizing Melanoma Cells and Microglia

Table S1. List of antibodies utilized in the study.

Antibody [clone]	Catalogue no.	Origin[1]	Application	Concentration	Manufacturer details
Polyclonal Anti-AIF-1/Iba1	NB100-1028	Goat	FACS	1:50	Novus Biologicals, Centennial, CO, USA
Polyclonal Anti-β-tubulin	ab6046	Rb	WB	1:1000	Abcam, Cambridge, MA, USA
Monoclonal Anti-CD16, REAfinity™ [REA423 | 3G8]	130-113-951	Ms	FACS	1:50	Miltenyi Biotec, Bergisch Gladbach, Germany
Monoclonal Anti-CD32 [2E1]	130-127-008	Ms	FACS	1:50	Miltenyi Biotec, Bergisch Gladbach, Germany
Monoclonal Anti-CD86, REAfinity™ [REA968 | 2331]	130-116-264	Ms	FACS	1:50	Miltenyi Biotec, Bergisch Gladbach, Germany
Monoclonal Anti-CD150 (SLAM), REAfinity™ [REA151 | A12]	130-099-722	Ms	FACS	1:50	Miltenyi Biotec, Bergisch Gladbach, Germany
Monoclonal Anti-CD163, REAfinity™ [REA812 | GHI/61.1]	130-112-287	Ms	FACS	1:50	Miltenyi Biotec, Bergisch Gladbach, Germany
Monoclonal Anti-CD206 [DCN228]	130-124-012	Ms	FACS	1:50	Miltenyi Biotec, Bergisch Gladbach, Germany
Monoclonal Anti-CH25H [1G8]	SC-293256	Ms	WB	1:500	Sana Cruz Biotechnology, Inc., Dallas, TX, USA
IgG1 isotype control [11711]	MAB002	Ms	Inhibition assay	1 μg/ml	R&D Systems, Minneapolis, MA, USA
Monoclonal IL-6Rα [17506]	MAB227	Ms	Inhibition assay	1 μg/ml	R&D Systems, Minneapolis, MA, USA
Monoclonal Anti-JunB [C37F9]	3753	Rb	WB	1:1000	Cell Signaling Technology, Inc., Danvers, MA, USA
Monoclonal Anti-PD-L1 [MIH2]	393602	Ms	FACS	1:50	BioLegend, San Diego, CA, USA
Polyclonal Anti-S1P1	ab11424	Rb	WB<br>FACS	1:1000<br>1:50	Abcam, Cambridge, MA, USA

[1]Ms: mouse, Rb: rabbit

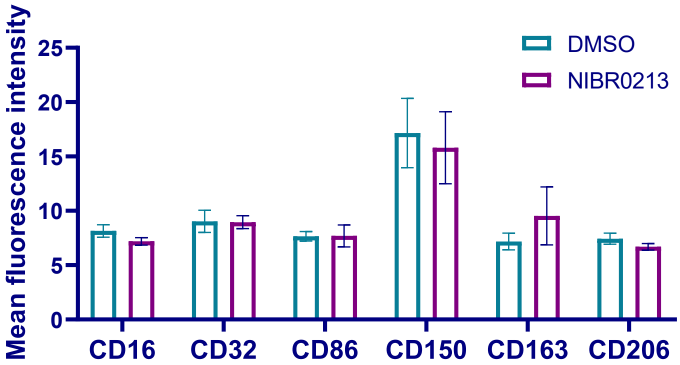

Figure S1. Mean fluorescence intensity (MFI) of inflammation-associated markers. CD16, CD32, CD86, CD150, CD163 and CD206 expression in NIBR0213- or DMSO-treated microglia cells was determined by flow cytometry. Bars represent the mean fluorescence intensity + SEM.
